# Effects of Dietary Supplementation With *Clostridium butyricum* on the Amelioration of Growth Performance, Rumen Fermentation, and Rumen Microbiota of Holstein Heifers

**DOI:** 10.3389/fnut.2021.763700

**Published:** 2021-11-11

**Authors:** Yang Li, Yiqiang Wang, Jingyi Lv, Xiujing Dou, Yonggen Zhang

**Affiliations:** College of Animal Sciences and Technology, Northeast Agriculture University, Harbin, China

**Keywords:** heifer, *Clostridium butyricum*, growth performance, rumen fermentation, rumen microbiota

## Abstract

In China, the use of antibiotics growth promoters as feed additives has been banned. The goal of raising dairy heifers is to gain a relatively high body weight on a high-fiber diet at first mating or calving, thus increasing economic benefits. The objective of this experiment was to explore the effects of supplemental *Clostridium butyricum* (*C. butyricum*) on growth performance, rumen fermentation and microbiota, and blood parameters in Holstein heifers. Twenty Holstein heifers [mean ± standard deviation (SD); age = 182 ± 4.20 d, body weight = 197.53 ± 5.94 kg, dry matter intake (DMI) = 6.10 ± 0.38 kg] were randomly assigned to one of two diets group for a 42-day feeding period: (1) basal diet (an untreated control group, i.e., the CON group) or (2) basal diet plus daily 2 × 10^8^ (colony-forming unit, CFU) of *C. butyricum* per kg of DMI per heifer (the CB group). The results demonstrated that *C. butyricum* supplementation increased the average daily gain from d 21 to 42 and DMI compared to the control group. Supplementation with *C. butyricum* significantly decreased the molar proportion of acetate and the acetate to propionate ratio but increased the molar proportion of butyrate and propionate. Compared with the control group, the relative abundance of *Butyrivibrio fibrisolvens, Ruminococcus albus, Ruminobacter amylophilus, Ruminococcus flavefaciens*, and S*treptococcus bovis* increased during the trial period in the CB group. However, *C. butyricum* had no significant effect on the blood parameters in Holstein heifers. In conclusion, these results show that feeding *C. butyricum* can improve growth performance and rumen fermentation without any negative impact on blood parameters in Holstein heifers.

## Introduction

The heifer stage is a vigorous period of growth and development for dairy cows because muscles, bones, and organs grow rapidly during this period. Cultivation at this stage is not only related to the development of the quality of the cow's body and the normal performance of lactation performance (1) but also consumes many costs. Raising dairy heifers aims to achieve a relatively high body weight gain with high-fiber diet at first mating or calving, thus increasing economic benefits (2). The use of antibiotics has been used in the past to improve their growth performance; however, with the ban of antimicrobial feed additives in china due to the problem of antibiotic residues and environmental pollution, there has been an increasing interest by ruminant nutritionists to find substitutes for antibiotics. Many microbial species have been approved as feed additives, such as *Clostridium butyricum*, which can improve digestibility and growth performance by improving intestinal health (3–6).

*Clostridium butyricum* (*C. butyricum*) is a gram-positive endophytic bacterium with anaerobic probiotics properties and can produce short-chain unsaturated fatty acids, especially butyric acid (7). A key feature of this species is that it can produce endospores, unlike *Lactobacillus* and *Bifidobacterium*, as well as survive relatively high bile concentrations and low pH (8), hence increasing their survivability in the rumen. Currently, *C. butyricum* is widely used in the production of aquatic and monogastric animals. Several studies have shown that supplementation with *C. butyricum* can improve the growth performance of kuruma shrimp (9), *Miichthys miiuy* (10) and tilapia (4), increasing their antioxidant or immune capacity. It has also been used as a dietary probiotic to benefit immune function and, more importantly, regulate the balance of intestinal flora in broiler chickens (11). In addition, the supplementation of less digestible diets with *C. butyricum* in weaned piglets has been shown to influence their growth positively (12). Therefore, we envisage that *C. butyricum* will have a similar positive effect on heifers fed high-fiber diets. Research on *C. butyricum* in dairy cows has focused mainly on immune regulation, milk composition improvement and milk production (13, 14); however, there are few studies on growth and development indicators for growth stages, such as in calves and heifers. Studies have shown that microbial feed additives such as yeast can improve the productive performance of ruminants by improving the activity and growth rate of rumen microorganisms (15). We hypothesize that *C. butyricum* can also affect rumen fermentation by adjusting the relative abundance of rumen microbiota, thereby improving the growth performance of heifers. Therefore, the objective of this study was to evaluate the effects of dietary supplementation with *C. butyricum* on growth performance, rumen fermentation, rumen microbiota and blood parameters in Holstein heifers.

## Materials and Methods

The Animal Care Advisory Committee of the Northeast Agricultural University approved all animal procedures and uses (protocol number: NEAU-[2011]-9, Harbin, China).

### Animals, Experimental Design, and Diets

Twenty Holstein heifers [mean ± standard deviation (SD); age = 182 ± 4.20 d, body weight (BW) = 197.53 ± 5.94 kg, dry matter intake (DMI) = 6.10 ± 0.38 kg] were blocked into 10 groups based on BW, DMI and age, and heifers within a block were randomly allocated to one of two diets group (10 calves per group): (1) an untreated control group (the CON group) and (2) a group treated daily with 2 × 10^8^ CFU per kg of DMI per heifer (the CB group). The *C. butyricum* LXKJ-1 was provided by Hubei Greensnow Biological Biotechnology Co., Ltd. (Wuhan, China; patent number: ZL 2016 1 0927003. 9), preservation number is CCTCC NO. M 2016130 in the China Center for Type Culture Collection, and the bacterial concentration reached 1 × 10^9^ CFU/g. Before the morning feeding, *C. butyricum* LXKJ-1 (2 × 10^8^ CFU/kg DMI) was individually hand - mixed with 200 g of the total mixed ration (TMR) feed, and the other was not (control). The ingredients and nutritional composition of the diet are given in Table 1. Diet [forage: concentrate = 50: 50, dry matter (DM) basis] was compounded according to the NRC recommendations (2001) to meet the nutrient requirements of heifers. Each heifer was individually kept in a tie stall pen in a barn, fed twice daily for at 06:00 and 18:00 with free access to water throughout the 42-day feeding trial. Based on the feed intake of the cow the day before, the amount of feed offered was adjusted daily to allow for at least 5% refusal (on an as-fed basis). The feed was pushed up at least 10 times per day.

**Table 1:** Composition and nutrient levels (g/kg dry matter) of experimental diets.

**Ingredients**	**Content (g/kg DM)**
Chinese wildrye	125.0
Alfalfa hay	165.0
Corn silage	210.0
Corn	208.6
Wheat bran	98.7
Soybean meal	39.7
DDGS^a^	33.3
Cottonseed meal	40.3
Rice hull powder	42.9
Rumen – protected fat^b^	16.5
Premix^c^	20.0
total	1,000.0
Nutrient levels	
DM	896.4
NE${}_{\text{L}}^{\text{d}}$, Mcal/kg DM	1.41
CP	153.5
EE	42.8
NDF	365.6
ADF	222.1
Ash	104.3
Ca	9.2
P	4.6

*DM, dry matter; CP, crude protein; EE, ether extract; NDF, neutral detergent fiber; ADF, acid detergent fiber; Ca, calcium; P, phosphorus.*

*^a^DDGS, dried distillers grains with solubles.*

*^b^Rumen – protected fat was palm oil fatty acids containing 985 g/kg EE.*

*^c^Premix provided the following per kg of diet: VA 30 150 IU, VD 9 675 IU, VE 76.5 mg, Fe 132 mg, Cu 26 mg, Mn 118.30 mg, Zn 166 mg, Se 0.07 mg, I 0.11 mg, Co 0.03 mg, Ca 6.76 g, and P 0.68 g.*

*^d^NE_L_ was a calculated value, while other nutrient levels were measured values*.

### Sample Collection and Laboratory Analysis

The experiment began when heifers were 6 months of age (Day 0). BW and DMI were measured on days 0, 1, 2, then on days 20, 21, 22 and finally on days 40, 41, 42. DMI was obtained by recording the weight of offered and refusal diet of individual heifers. The average daily gain (ADG), [(kg of final BW – kg of initial BW)/experimental days] and feed efficiency (kg of ADG/kg of the DMI) were then calculated (16).

Before the morning feeding, blood samples were collected from the jugular vein using 10-mL evacuated blood-collection tubes containing heparin on days 0, 1, 2, then on days 20, 21, 22 and finally on days 40, 41, 42, and were centrifuged at 3,000 × *g* at 4°C for 10 min. Plasma was collected and stored at −40°C for further analysis. Plasma concentrations for blood urea nitrogen (BUN), cholesterol (CHOL), glucose (GLU), triglyceride (TG), and total protein (TP) levels were determined on a fully automated biochemical analyzer using standard commercial kits supplied from Biosino Bio-tec (Beijing, China). Concentrations of catalase (CAT), glutathione peroxidase (GSH-PX), malondialdehyde (MDA), total antioxidant capacity (T-AOC), and total superoxide dismutase (T-SOD) in plasma were determined by colorimetry using standard commercial kits supplied from Nanjing Jian Cheng Bioengineering Institute (Nanjing, China). Plasma concentrations of immunoglobulin A (IgA), immunoglobulin G (IgG) and immunoglobulin M (IgM) were analyzed using commercial ELISA kits (Abnova Corporation, Taipei, Taiwan, China). Three hours after the morning feeding on d 0, 1, 2, 20, 21, 22, 40, 41, and 42 of the experiment, a stomach tube equipped with a 200-mL syringe (Shanghai Syringe Factory Sales Company, Shanghai, China) was used to collect rumen fluid samples from each heifer. To prevent saliva contamination during rumen fluid collection, the first 100 ml of liquid collected was discarded. The collected rumen fluid was filtered through 4 layers of cheesecloth, and the pH was immediately measured using a pH meter (Sartorius Basic pH Meter, Germany). An aliquot (5 mL) of rumen filtrate was acidified with 1 mL of 250 g/kg metaphosphoric acid and stored at −20°C for analysis of ammonia-N (NH_3_-N), volatile fatty acid (VFA), and microbial crude protein (MCP) concentrations. The VFA concentration was measured by gas chromatography (GC-8A; Shimadzu Corp., Kyoto, Japan) (17). The Ammonia-N was determined using the phenol/hypochlorite method (18), while the ruminal MCP concentration was determined using the spectrophotometric method (19).

Total DNA were extracted from the rumen contents by the modified bead-beating protocol (20). The real-time PCR was carried out using a real-time PCR machine (ABI PRISM 7500 SDS thermal cycler, Applied Biosystems, Foster City, CA, USA) using SYBR Green Supermix (TaKaRa Biotechnology Co., Ltd. Dalian, China). All the operations were carried out according to the manufacturer's instructions. The PCR primer sets used are shown in Table 2. The group-specific primers for total bacteria (reference genes) and species-specific primers for *Butyrivibrio fibrisolvens* (*B*. *fibrisolvens*), *Fibrobacter succinogenes* (*F*. *succinogenes*), *Prevotella ruminicola* (*P*. *ruminicola*), *Ruminococcus albus* (*R*. *albus*), *Ruminobacter amylophilus* (*R*. *amylophilus*), *Ruminococcus flavefaciens* (*R*. *flavefaciens*), and *Streptococcus bovis* (*S*. *bovis*) were designed according to the methods described previously (21, 22). Relative gene expression of microbes was calculated using the 2^−ΔΔCt^ method as follows: Relative quantification = 2^−[(Cttargetgene−Ctreferencegene)treatmentgroup−(Ct^^*targetgene*−*Ctreferencegene*)*controlgroup*]^ (23), where Ct represents the threshold cycle.

**Table 2:** Primers used for RT-PCR detection of microbial species.

**Target species tested**	**Forward primer**	**Size (bp)**
	**Reverse primer**	
Total bacterial^a^	CGGCAACGAGCGCAACCC	130
	CCATTGTAGCACGTGTGTAGCC	
*B. fibrisolvens* ^b^	ACCGCATAAGCGCACGGA	65
	CGGGTCCATCTTGTACCGATAAAT	
*S. bovis* ^b^	TTCCTAGAGATAGGAAGTTTCTTCGG	127
	ATGATGGCAACTAACAATAGGGGT	
*R. amylophilus* ^b^	CTGGGGAGCTGCCTGAAT	100
	CATCTGAATGCGACTGGTTG	
*P. ruminicola* ^b^	GCGAAAGTCGGATTAATGCTCTATG	78
	CCCATCCTATAGCGGTAAACCTTTG	
*R. flavefaciens* ^b^	CGAACGGAGATAATTTGAGTTTACTTAGG	132
	CGGTCTCTGTATGTTATGAGGTATTACC	
*R. albus* ^b^	CCCTAAAAGCAGTCTTAGTTCG	176
	CCTCCTTGCGGTTAGAACA	
*F. succinogenes* ^b^	GGAGCGTAGGCGGAGATTCA	97
	GCCTGCCCCTGAACTATCCA	

*R. flavefaciens, Ruminococcus flavefaciens; R. albus, Ruminococcus albus; F. succinogenes, Fibrobacter succinogenes; B. fibrisolvens, Butyrivibrio fibrisolvens; S. bovis, Streptococcus bovis; P. ruminicola, Prevotella ruminicola; R. amylophilus, Ruminobacter amylophilus.*

*^a^Denman and Mcsweeney (2009).*

*^b^Khafipour et al. (2009)*.

### Statistical Analyses

Data were analyzed using SAS software (version 9.4, SAS Institute Inc., Cary, NC). Data on ADG and feed efficiency were analyzed using the one-way ANOVA procedure with *C. butyricum* treatment used as the main factor. Data on growth performance, DMI, plasma parameters, rumen fermentation parameters and microbes were analyzed using the PROC MIXED program of SAS software. A randomized block design with repeated measures was used, with time, treatment, and interaction of treatment × time as fixed effects and cow within treatment as a random effect. The data obtained from 0 day were added to the model as covariates in the statistical analysis. The level of significance was set at *P* <0.05, and differences were considered statistical trends when 0.05 < *P* ≤ 0.10. Standard errors of the mean are reported.

## Results

### Effects of *Clostridium butyricum* on Growth Performance

As shown in Table 3, BW (231.41 vs. 233.93 kg; *P* = 0.048) increased with the administration of *C. butyricum* and was influenced by time (*P* <0.0001) and treat × time (*P* = 0.0001). DMI was increased with increasing *C. butyricum* supplementation dose (6.76 vs. 7.12 kg; *P* <0.0001) and was influenced by time (*P* <0.0001), but not by treat × time (*P* = 0.66). *C. butyricum* supplemented diet significantly increased ADG from day 21 to 42 (1.14 vs. 1.22 kg; *P* = 0.0003) and there was no differences in feed efficiency between the different treatments over the entire test period.

**Table 3:** Effect of *Clostridium butyricum* on growth performance, feed efficiency, and average daily gain in heifers.

**Items**	**Treatment** ^**a**^		**SEM^**b**^**	***P*** **-value**		
	**CON**	**CB**		**Treat**	**Time**	**Treat × Time**
Body weight, kg	231.41	233.93	1.064	0.048	<0.0001	0.0001
Dry matter intake, kg/d	6.76	7.12	0.0483	<0.0001	<0.0001	0.66
Average daily gain_(d 0−21)_, kg	1.07	1.09	0.101	0.87	–	–
Average daily gain_(d 21−42)_, kg	1.14	1.22	0.0125	0.0003	–	–
Average daily gain_(d 0−42)_, kg	1.11	1.16	0.051	0.49	–	–
Feed efficiency_(d 0−21)_	0.17	0.17	0.0166	0.92	–	–
Feed efficiency_(d 21−42)_	0.17	0.17	0.00205	0.39	–	–
Feed efficiency_(d 0−42)_	0.17	0.17	0.00810	0.74	–	–

*^a^CON, control diet; CB = 2 ×10^8^ CFU/kg of dry matter intake.*

*^b^SEM, Standard error of the mean*.

### Effect of *Clostridium butyricum* on Plasma Parameters in Heifers

The results of plasma parameters are presented in Table 4. Within the experiment, no significant differences were observed on biochemical, antioxidant, and immunological levels in heifers between the two groups, and they were not affected by time and treat × time. However, glucose (6.13 vs. 6.28 mmol/L; *P* = 0.08) trended to increase with *C. butyricum* supplementation.

**Table 4:** Effect of *Clostridium butyricum* on plasma parameters in heifers.

**Items**	**Treatment** ^**a**^		**SEM^**b**^**	***P*** **-value**		
	**CON**	**CB**		**Treat**	**Time**	**Treat × Time**
**Biochemical levels**						
TP, g/L	63.76	65.62	0.824	0.12	0.63	0.32
BUN, mmol/L	3.54	3.27	0.178	0.28	0.26	0.16
GLU, mmol/L	6.13	6.28	0.0747	0.08	0.79	0.35
CHOL, mmol/L	2.86	3.17	0.144	0.15	0.97	0.42
TG, mmol/L	0.29	0.30	0.00927	0.27	0.77	0.41
**Antioxidant levels**						
T-AOC, mmol/ml	11.24	13.01	1.010	0.26	0.24	0.71
T-SOD, U/ml	78.84	79.03	2.221	0.95	0.61	0.66
MDA, nmol/ml	3.50	3.36	0.206	0.64	0.67	0.56
CAT, U/ml	58.30	50.74	4.555	0.21	0.53	0.81
GSH, U/ml	7.78	7.79	0.306	0.97	0.69	0.11
**Immunological levels**						
IgA, g/L	0.76	0.71	0.0313	0.34	0.31	0.34
IgG, g/L	9.94	10.32	0.284	0.37	0.19	0.65
IgM, g/L	2.51	2.58	0.0784	0.40	0.51	0.82

*TP, total protein; TG, triglyceride; CHOL, cholesterol; BUN, blood urea nitrogen; GLU, blood glucose; CAT, catalase; MDA, malondialdehyde; GSH-Px, glutathione peroxidase; T-SOD, total superoxide dismutase; T-AOC, total antioxidant capacity; IgA, immunoglobulin A; IgG, immunoglobulin G; IgM, immunoglobulin M.*

*^a^CON, control diet; CB = 2 ×10^8^ CFU/kg of dry matter intake.*

*^b^SEM, Standard error of the mean*.

### Effects of *Clostridium butyricum* on Ruminal Fermentation

Table 5 shows the results for ruminal pH, ammonia-N and VFA concentrations. There were no significant differences in pH, ammonia-N and MCP concentration with *C. butyricum* supplementation. The TVFA concentration was not affected by *C. butyricum* supplementation (53.91 vs. 54.10 mM; *P* = 0.75) but was influenced by time (*P* <0.0001). With increasing *C. butyricum* supplementation, molar proportion of ruminal propionic acid was increased (21.74 vs. 23.54 mol/100 mol; *P* = 0.001) and the acetate to propionate ratio were decreased (2.68 vs. 2.37; *P* = 0.0002) but time (*P* = 0.77 or *P* = 0.75, respectively) and treatment × time (*P* = 0.99 or *P* = 0.32, respectively) had no influence. Furthermore, the molar proportion of butyric acid increased (15.28 vs. 17.15 mol/100 mol; *P* <0.0001) with increasing *C. butyricum* supplementation and was affected by time (*P* = 0.0002) and treat × time (*P* <0.0001). In addition, the molar proportion of acetic acid was decreased (57.79 vs. 55.29 mol/100 mol; *P* = 0.0003) and was influenced by treat × time (*P* = 0.006).

**Table 5:** Effect of *Clostridium butyricum* on ruminal fermentation in heifers.

**Items**	**Treatment** ^**a**^		**SEM^**b**^**	***P*** **-value**		
	**CON**	**CB**		**Treat**	**Time**	**Treat × Time**
pH	6.85	6.87	0.0156	0.45	0.78	0.79
Ammonia-N, mg/dL	7.61	7.62	0.278	0.98	0.98	0.11
MCP^c^, mg/dL	110.22	109.21	1.985	0.72	0.16	0.35
Total VFA, mM	53.91	54.10	0.438	0.75	<0.0001	0.83
Acetate, mol/100 mol	57.79	55.29	0.413	0.0003	0.14	0.006
Propionate, mol/100 mol	21.74	23.54	0.349	0.001	0.77	0.99
Butyrate, mol/100 mol	15.28	17.15	0.137	<0.0001	0.0002	<0.0001
Acetate:propionate	2.68	2.37	0.0519	0.0002	0.75	0.32

*^a^CON, control diet; CB = 2 ×10^8^ CFU/kg of dry matter intake.*

*^b^SEM, Standard error of the mean.*

*^c^MCP, microbial crude protein*.

### Effects of *Clostridium butyricum* on Rumen Microbiota

The relative abundance of ruminal microbiota is presented in Table 6. The *C. butyricum* had no effect on the relative abundance of *F. succinogenes* and *P. ruminicola* in the CB group. And *C. butyricum* significantly increased the relative abundance of *R. flavefaciens* (1.03 vs. 1.91; *P* = 0.0001), *R. albus* (1.02 vs. 1.67; *P* = 0.04) and *S. bovis* (1.00 vs. 2.06; *P* = 0.003) and was influenced by time (*P* = 0.001, *P* = 0.003 or *P* <0.0001, respectively) and treat × time (*P* = 0.001, *P* = 0.003 or *P* <0.0001, respectively). The relative abundance of *B. fibrisolvens* and *R. amylophilus* increased (1.01 vs. 1.53; *P* = 0.02 or 1.00 vs. 1.53; *P* = 2.06, respectively) with *C. butyricum* supplementation and was not influenced by time (*P* = 0.68 or *P* = 0.89, respectively) and treat × time (*P* = 0.73 or *P* = 0.89, respectively).

**Table 6:** Effect of *Clostridium butyricum* on rumen microbiota in heifers.

**Items**	**Treatment** ^**a**^		**SEM^**b**^**	***P*** **-value**		
	**CON**	**CB**		**Treat**	**Time**	**Treat × Time**
*F. succinogenes*	1.03	1.25	0.112	0.21	0.58	0.43
*R. flavefaciens*	1.03	1.91	0.138	0.0001	0.001	0.001
*R. albus*	1.02	1.67	0.218	0.04	0.003	0.003
*B. fibrisolvens*	1.01	1.53	0.148	0.02	0.68	0.73
*P. ruminicola*	1.02	1.30	0.128	0.14	0.31	0.30
*R. amylophilus*	1.07	3.60	0.663	0.01	0.89	0.89
*S. bovis*	1.00	2.06	0.218	0.003	<0.0001	<0.0001

*R. flavefaciens, Ruminococcus flavefaciens; R. albus, Ruminococcus albus; F. succinogenes, Fibrobacter succinogenes; B. fibrisolvens, Butyrivibrio fibrisolvens; S. bovis, Streptococcus bovis; P. ruminicola, Prevotella ruminicola; R. amylophilus, Ruminobacter amylophilus.*

*^a^CON, control diet; CB = 2 ×10^8^ CFU/kg of dry matter intake.*

*^b^SEM, Standard error of the mean*.

## Discussion

Under the premise of being healthy, obtaining the highest weight at the lowest cost is one of the most important goals when heifers are at the age of first mating or calving. With the gradual withdrawal of feed antibiotics, probiotic feed additives have become increasingly popular. The increase in the number of beneficial bacteria in the feces, improvement of intestinal histology, and the enhancement of intestinal digestive enzyme activity may all be associated to the potential of *C. butyricum* HJCB998 to enhance the intestinal absorption capacity of animals, thereby improving growth performance (3). Studies for monogastric (11, 24) and ruminant (6, 14) have confirmed the positive effects of *C. butyricum* on the production performance and total tract apparent digestibility. Therefore, although apparent digestibility was not measured in this study, the positive effect of *C. butyricum* LXKJ-1 on growth performance can be inferred from the increasing in ADG and DMI of dairy heifers. Although the digestive system of monogastric animals is different from that of ruminants, a large number of positive research results on monogastric animals has given us confidence in its application in ruminants. In a related study, the feed-gain ratio of pig was reduced with *Clostridium butyricum* UCN - 12 supplementation, which could translate into reduce feed costs during production (12). The above studies provide theoretical references for *C. butyricum* LXKJ-1 regarding increased body weight in heifers. In this experiment, *C. butyricum* LXKJ-1 significantly increased the DMI, ADG and BW of heifers. In addition, the results showed that the improvement of rumen fermentation by *C. butyricum* LXKJ-1 may provide more energy for the growth of heifers. *C. butyricum* could produce a large amount of short-chain fatty acids during anaerobic fermentation, including propionic acid and butyric acid (25) which serves as energy source for cells. Therefore, *C. butyricum* UCN-12 not only increases the molar proportion of propionic acid in the rumen but is also a butyric acid-producing probiotic typically implicated in the production of butyric acid (5). As specific nutrients and energy components, propionic acid and butyric acid could also provide more energy for heifers, thereby improving their growth performance.

For heifers, ensuring the health and improving growth performance are equally important. Plasma biochemical indicators can reflect the body health condition and metabolic level of heifers; therefore, for the application of new feed additives, it is essential to verify the effects of additives on blood biochemical indicators (26). BUN is an indicator of protein and amino acid metabolism in the body. TP reflects the protein absorption and reflects the level of immunity (27). The content of TG and CHOL in plasma is also an essential indicator of the blood lipid level of the animal. In this study, the addition of *C. butyricum* LXKJ-1 in the diet did not affect heifers' protein and fat metabolism. However, the increasing trend of blood GLU levels were observed after feeding *C. butyricum* LXKJ-1 related to increased molar proportion of propionate, a glucogenic precursor formed in the rumen and increase blood glucose availability via gluconeogenesis in the liver (28). The antioxidant system can prevent animals from being harmed by free radicals and environmental stimuli generated. Enhancing the immune response can promote the improvement of the disease resistance of the animal body and improve the growth performance. Antioxidant enzyme activity and immunoglobulin content are essential indicators that reflect the body's antioxidant capacity and immune function. Kohiruimaki et al. (13) found that adding *C. butyricum Miyairi* 588 can enhance the number of CD_4+_ T cells and improve the immunity of transition dairy cows. However, in this experiment, *C. butyricum* LXKJ-1 did not seem to affect the antioxidant and immune functions of heifer. The difference between the results of this experiment and previous studies may be due to by differences in *C. Clostridium* species, animal species and experimental period. Considering the importance of *C. butyricum* LXKJ-1 to improve the antioxidant and immune capacity of animals to replace feed antibiotics, the efficacies of *C. butyricum* LXKJ-1 to improve immunological functions need further investigation.

After verifying the safety of new feed additives, we want to further unravel the reason why C. butyricum LXKJ-1 improved the performance of heifers. For ruminants, VFAs are the main source of energy. Therefore, it is necessary to deeply study the influence of *C. butyricum* LXKJ-1 on rumen fermentation and rumen microbiota of heifer. The improvement of rumen fermentation and the regulation of the relative numbers of cellulolytic bacteria and amylolytic bacteria by *C. butyricum* LXKJ-1 in the rumen are important findings of this experiment. We found that total VFAs concentration in the rumen is affected by time, which may be related to the increase in DMI with the extension of the experimental period. It has been previously reported that feeding *C. butyricum* to dairy cows affected the production of VFAs in the rumen (14); moreover, previous experiments have suggested that microbial feed additives can affect rumen VFA production by adjusting the number of rumen microbes (26, 29). In this experiment, supplementation with *C. butyricum* LXKJ-1 significantly increased the number of several major cellulolytic bacteria and amylolytic bacteria and had a greater impact on the relative abundance of two amylolytic bacteria *S*. *bovis* and *R*. *amylophilus*. This is likely the main reason why *C. butyricum* LXKJ-1 could influence rumen fermentation and increase the molar proportion of ruminal propionic acid in heifers. In addition, we found that *C. butyricum* LXKJ-1 decreased the molar proportion of acetic acid and the ratio of acetate to propionate in the rumen, which may be caused by the increase in the propionic acid production. At the same time, we also found that supplementation with *Clostridium butyricum* also significantly improved relative abundance of *R. flavefaciens, R. albus*, and *B*. *fibrisolvens*. There were time or treatment × time effects for total VFA concentration, the molar proportion of butyric acid and the relative abundance of *R. flavefaciens, S. bovis, R. albus* as expected in growing heifers. The significant effects of time and interaction demonstrated the continuous effect of *C. butyricum* LXKJ-1 on rumen fermentation and relative abundance of microbiota. The regulatory mechanism of *C. butyricum* LXKJ-1 on the number of rumen microbes is still unclear. However, there are reports in the literature that *C. butyricum* can regulate the number of bacteria in the intestine and feces in broiler chickens (*C. butyricum* HJCB998) (11), sows (*C. butyricum* UCN-12) (5) and tilapia (China Center for Type Culture Collection accession NO. M2014537) (4). Some microbial feed additives contain different enzymes, vitamins, and some unidentified cofactors that may enhance the microbial activity and growth rate in the rumen (15). *C. butyricum*, in addition to the production of short-chain fatty acids during metabolism, also produces some nutritional factors, such as enzymes (exo-pectate lyase, pectin methylesterase, and endo-pectate lyase) and vitamins (vitamin B and E), which may provide favorable conditions for the growth of rumen microorganisms (30–32). Therefore, the regulatory mechanism of *C. butyricum* LXKJ-1 impact on rumen microbes needs further study. In addition, increase in the relative expression of rumen bacteria may also increase the ruminal degradation of protein and carbohydrates in the diet (33), thereby increasing total tract apparent digestibility (6, 26).

**Figure 1:**
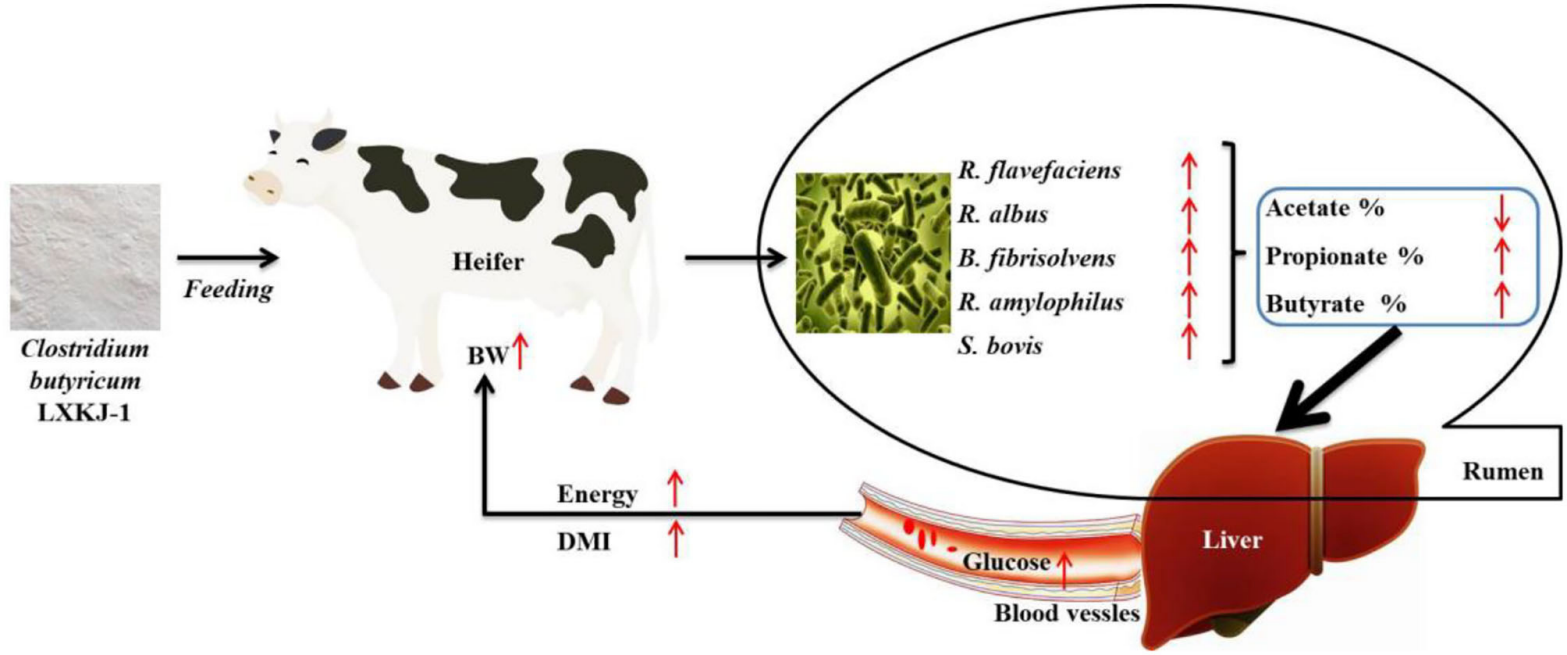
Pathway of *Clostridium butyricum* regulation of rumen fermentation to improve the growth performance of heifer.

In summary, as shown in Figure 1, the research indicates that *C. butyricum* LXKJ-1 can improve the rumen fermentation parameters by adjusting the number of rumen microbiota, thereby improving the growth performance of the heifers. Therefore, this study provides a theoretical grounding for enhancing the growth performance of heifers by *C. butyricum* supplements.

## Conclusions

Dietary supplementation with *C. butyricum* could increase BW, DMI and enhance the rumen fermentation functions by increasing the abundance of rumen microbiota and improving molar proportion of propionate and butyrate without any negative impact on blood parameters in heifers. Under the experimental conditions, *C. butyricum* is an effective microbial feed additive that could be used in the production of heifers.

## Data Availability Statement

The original contributions presented in the study are included in the article/supplementary material, further inquiries can be directed to the corresponding authors.

## Ethics Statement

All animal procedures and uses were approved by the Animal Care Advisory Committee, Northeast Agricultural University.

## Author Contributions

YL and YW conceived, designed the experiments, and wrote the paper. YW, JL, and YL conducted the experiments. XD and YZ supervised the work. All authors reviewed the manuscript.

## Funding

This study was financially supported by the University Nursing Program for Young Scholars with Creative Talents in Heilongjiang Province (UNPYSCT-2020095).

## Conflict of Interest

The authors declare that the research was conducted in the absence of any commercial or financial relationships that could be construed as a potential conflict of interest.

## Publisher's Note

All claims expressed in this article are solely those of the authors and do not necessarily represent those of their affiliated organizations, or those of the publisher, the editors and the reviewers. Any product that may be evaluated in this article, or claim that may be made by its manufacturer, is not guaranteed or endorsed by the publisher.
